# The Preparation, Antioxidant Activity Evaluation, and Iron-Deficient Anemic Improvement of Oat (*Avena sativa* L.) Peptides–Ferrous Chelate

**DOI:** 10.3389/fnut.2021.687133

**Published:** 2021-06-21

**Authors:** He Yuanqing, Yang Pengyao, Ding Yangyang, Chen Min, Guo Rui, Duan Yuqing, Zhang Haihui, Ma Haile

**Affiliations:** ^1^College of Food Science and Biological Engineering, Jiangsu University, Zhenjiang, China; ^2^The Laboratory Animal Research Center, Jiangsu University, Zhenjiang, China; ^3^School of the Environment and Safety Engineering, Jiangsu University, Zhenjiang, China

**Keywords:** iron supplement, peptides-ferrous chelate, oat peptide, iron-deficient anemic, antioxidant activity

## Abstract

Iron-chelating peptides have been widely considered as one of the best iron supplements to alleviate the iron deficiency. In this study, a novel oat peptides–ferrous (OP-Fe^2+^) chelate was prepared from antioxidant oat peptides obtained in the laboratory of the authors. The optimal preparation condition was obtained through the single-factor and response surface methodology, and the chelating rate could reach up to 62.6%. After chelation, the OP-Fe^2+^ chelate exhibited a significantly higher 2,2-diphenyl-1-picrylhydrazyl radical scavenging activity than oat peptides. It was discovered that the hemoglobin concentration and the number of red blood cell levels in OP-Fe^2+^-treated iron-deficient anemic (IDA) rats were significantly higher than untreated IDA rats. The OP-Fe^2+^ chelate could also improve the hypertrophy of the spleen, serum iron (SI), total iron and binding capacity, and serum ferritin levels in the IDA rats. In addition, the OP-Fe^2+^ treatment significantly increased the antioxidant activities of super oxidase and glutathione in the liver homogenate of the IDA rats. Therefore, the OP-Fe^2+^ chelate is an effective type of iron supplement for IDA rats, which could be a promising source with anti-anemia and antioxidant activity.

**Graphical Abstract:**
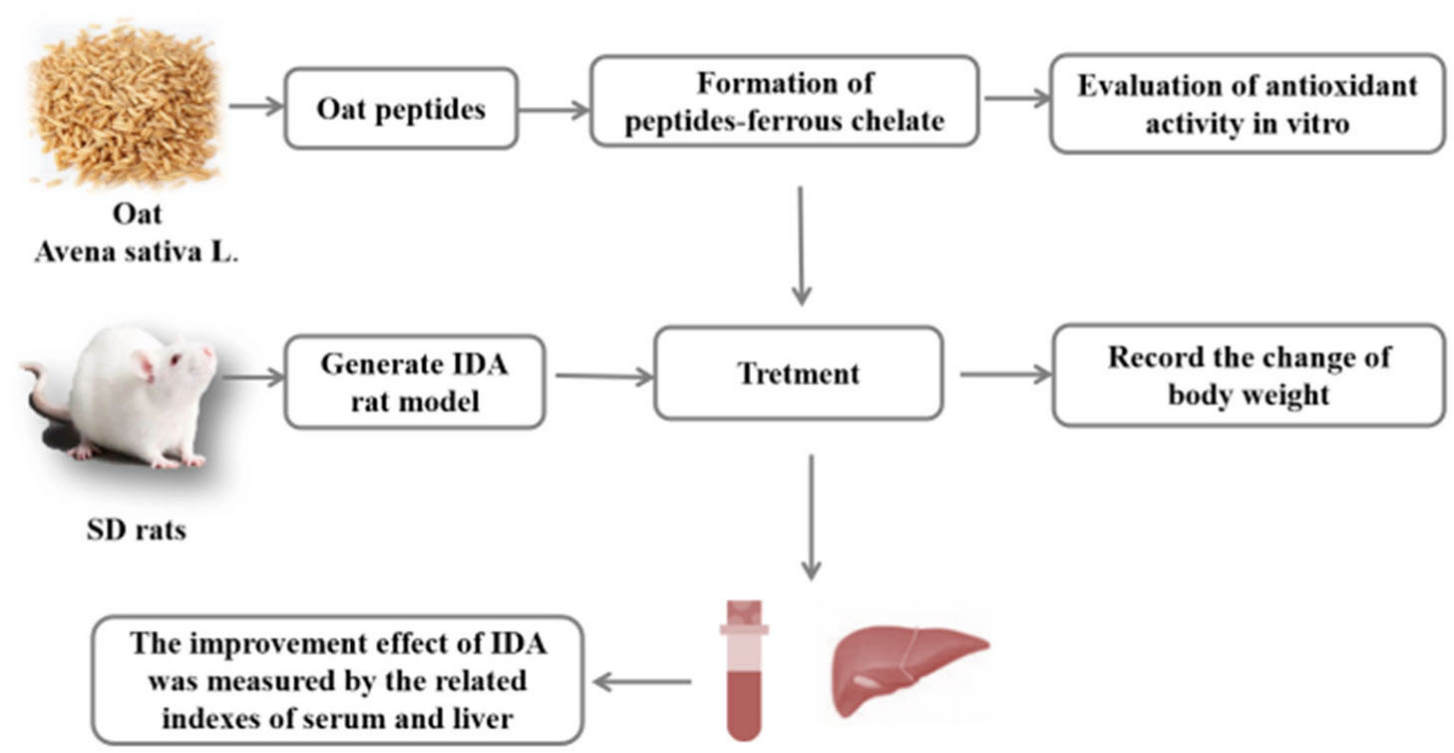
An overview of the experiment. In this study, OP-Fe^2+^ was prepared from the antioxidant oat peptides. After chelation, the antioxidant activities of OP-Fe^2+^ is higher than OP. In addition, animal experiments indicated that OP-Fe^2+^ can be used as an effective iron supplement and antioxidant.

## Highlights

- After chelation, oat peptides-ferrous chelate (OP-Fe^2+^) exhibited a signif-icantly higher 2, 2-diphenyl-1-picrylhydrazyl (DPPH) radical scavengingactivity than oat peptides (OP).
- OP-Fe^2+^ chelate showed a good iron supplemental effect on IDA rats.
- OP-Fe^2+^ was helpful to restore the antioxidant capacity of anemic rats.
- OP-Fe^2+^ chelate could avoid the body damage caused by the iron deficiency anemia.

## Introduction

Iron, a kind of essential trace element for humans and animals, participates in multiple biological processes, such as protein and DNA synthesis (1). Iron works as a vital part of cytochrome, hemoglobin, and enzymes, and plays an important role in transporting and storing oxygen for breathing and metabolism (2). Insufficient iron absorption or iron loss from dietary sources can lead to iron deficiency (1). Iron deficiency may cause anemia, with common symptoms, such as fatigue, dizziness, and irritability. Iron deficiency anemia has been regarded as a global problem that troubles one third of the global population, particularly infants, pregnant women, and the elderly (3). Iron deficiency anemia affects more than 1.2 billion individuals worldwide, and iron deficiency in the absence of anemia is even more frequent (4). The most common manifestations are pregnancy accidents as well as developmental delay and cognitive impairment in young children. Iron deficiency could result in a series of dysfunctions, such as chronic fatigue and weakened immune function in adults (5).

Many iron supplements have been exploited to solve this emergency problem. The use of inorganic ferrous-iron salt has been limited by side effects and low bioavailability (6, 7). Recently, bioactive peptides derived from food protein as a kind of new metal ion chelating agent have attracted the interest of people (8). Peptide-Fe^2+^, with greater bioactivity, high absorption, and no side effects has been considered as one of the best iron supplements to improve iron deficiency (9). Some ferrous-iron chelating peptides have been discovered and characterized, which can be used as iron supplements. For example, ferrous-iron-chelating peptides from Alaska pollock frames (APFP-Fe) are a useful iron source for improving iron nutritional status in iron deficiency anemic rats (10). Chickpea peptides, through metal chelation, may increase iron solubility and bioavailability, and improve iron absorption (11). Hairtail protein hydrolysate, through metal chelation, showed an anti-fatigue effect (12). The peptide from barley protein could enhance the level of iron absorption and ferritin in Caco-2 cells (8). However, little attention has been paid to the function of the ferrous-iron-chelating peptide from oat.

As we all know, oat has the highest amount of proteins among cereals, which is low price and easy to obtain (13). In addition, the biological value (64.9%), net protein utilization (65.7%), and protein efficiency ratio (2.25) of oat protein are significantly higher compared with other cereals (14). These data mean that the oat protein will have a higher application prospect than other plant proteins. Oat bioactive peptides have been proved to have a good antioxidant activity (8), and hypotensive and hypoglycemic activities (15, 16). Karas et al. (17) also found that oat peptides are structurally similar to epidermal cell growth factor (EGF), which could be easily absorbed by the skin, and then improve skin metabolism. The application of oat peptides is significant for the protection of human health. Oat bioactive peptides may be used to prepare OP-Fe^2+^, which could increase chelation efficiency. Moreover, oat bioactive peptides could prevent Fe^2+^ oxidation in the gastrointestinal tract after being ingested, and increase the bioavailability of Fe^2+^. The purpose of this study was to obtain the best conditions for the preparation of OP-Fe^2+^ and confirm its iron supplement and antioxidative function by *in vitro* and *in vivo* methods.

## Materials and Methods

### Materials

Oat was purchased from Shijiazhuang Lingfeng Agricultural and Sideline Products Development Co., Ltd. (Hebei, China). Alkaline protease with a reported activity of 2.4 AU g^−1^ was purchased from Novozym Biotechnology Co., Ltd. (Henan, China). Ferrous chloride (molecular weight 198.83) was obtained from Sinopharm Chemical Reagent Co., Ltd. (Shanghai, China). Other chemical reagents used in this study were of analytical grade and commercially available.

### Preparation of Oat Peptides

The oat peptides were prepared and obtained in the laboratory of the authors. Oat protein was obtained through methods of alkaline extraction and acid precipitation. The hydrolysis reaction was proceed using alkaline protease under optimized conditions as follows: 2.5% (wt/wt, defined as enzyme mass/substrate mass × 100%), 9.5 (pH), 55°C (temperature) and 2 h (time). Then, the hydrolysate was heated at 90°C for 15 min to inactivate the enzyme followed by centrifugation at 1,350 × g for 20 min at room temperature to obtain the supernatant. The fraction with formula weight <3 kDa in the hydrolysate was collected with the ultra-filtration membrane (3 kDa). After freeze-drying, the obtained oat peptides (OPs) were stored at −20°C for the next step.

### Preparation of Peptides–Ferrous

Single-factor tests were performed to optimize chelating conditions (18). First, OP solutions with different concentrations (2, 3, 4, 5, and 6%) were prepared, and 0.5% ascorbic acid was added in the solution to prevent the oxidation of Fe^2+^. Then, FeCl_2_ was added to form OP-Fe^2+^ with different mass ratios in OP and FeCl_2_ (OP-FeCl_2_ ratios: 1:1, 2:1, 3:1, 4:1, 5:1, and 6:1). The mixture was incubated in water bath equipment at different pH (3, 4, 5, 6, and 7) and temperature (20, 30, 40, 50, and 60°C) for different lengths of time (20, 30, 40, 50, and 60 min). After chelation, the supernatant was collected after centrifugation at 1,350 × g for 10 min followed by the addition of 95% ethanol to the supernatant (ethanol: supernatant = 6:1). The mixed solution was left to settle statically for 1 h. The sediment (OP-Fe^2+^) was collected after centrifugation at 6,010 × g for 20 min and then washed with 95% ethanol 5 times to remove excess Fe^2+^. The OP-Fe^2+^ powder was collected after the sediment was freeze-dried. The chelating ratio (CR) was calculated by the following formula:

$$\begin{array}{l} CR=\left(B/A\right)\times \;100\% \end{array}$$

where, A (mg) represents total content of Fe^2+^ in the reaction system; B (mg) represents the content of Fe^2+^ in the OP-Fe^2+^chelate.

### Experiment Design of Response Surface Methodology

The ranges of the factors were determined based on the results of the single factor experiment. A Box–Behnken experimental design with three factors and three levels was chosen to optimize the chelation conditions of OP-FeCl_2_. The chelating rate was used as the response value. Factors and levels designed for RSM, such as pH (5, 6, 7), OP-FeCl_2_ ratio (3, 4, 5), and OP concentration (2, 3, 4%) are shown in Table 1.

**Table 1:** Factors and levels of RSM.

**Factors**	**Levels**		
	**−1**	**0**	**1**
(A) pH	5	6	7
(B) OP concentration	2	3	4
(C) OP-FeCl_2_ ratio	3	4	5

### Activity of Scavenging 2,2-diphenyl-1-picrylhydrazyl Radicals

The activity of scavenging DPPH radicals was measured according to the reference method (19). Sample solutions (1 ml) with different concentrations (6.25, 12.5, 25, 50 mg/ml) were blended with a 4-ml DPPH ethanol solution (0.12 mmol/L, prepared with 95% ethanol), and then kept in the dark for 30 min at 25°C. Absorbance was measured at 517 nm with a microplate spectrophotometer (Biotek Instruments, Winooski, VT, United States). The activity of scavenging DPPH radicals was calculated as follows:

$$\begin{array}{l} \text{The activity of scavenging }DPPH\;radicals\;(\%)=[1\\ \;-(A_{\text{i}}-A_{\text{j}})/A_{\text{C}}]\times \;100\%, \end{array}$$

(A_i_-A_j_) and A_C_ represent the absorbance of the sample and the control, respectively.

### Activity of Scavenging Hydroxyl Radicals

The activity of scavenging hydroxyl radicals was measured according to the following method: solutions with different concentrations of OP (3.125, 6.25, 12.5, 25, and 50 mg/ml) and OP-Fe^2+^ (3.125, 6.25, 12.5, 25, and 50 mg/ml) complex were prepared. Sample solutions (2 ml) with different concentrations blended with FeSO_4_ (0.5 ml, 9 mmol/L, prepared with ethanol), salicylic acid (0.5 ml, 9 mmol/L, prepared with ethanol), and H_2_O_2_ (0.5 ml, 0.15%) were incubated at 37°C for 30 min. Then, absorbance was measured at 510 nm.

### Animal Experiments and Experimental Design

The animal experiment was approved by the Institutional Animal Care and Use Committees of Jiangsu University (UJS-IACUC-2020072201). Sixty male Sprague–Dawley (SD) rats (No. 202009460) weighing 40–50 g were purchased from Nanjing Medical University [SCXK (SU) 2016-0002] and fed at the Laboratory Animal Research Center of Jiangsu University [SYXK (SU) 2018-0053, the temperature at 20–26°C, the humidity of 40–70%, the diurnal cycle of 12 h]. Rats achieved distilled deionized water in the period of the study freely. After adaptation for 3 days, the 60 rats were randomly assigned to six groups (*n* = 10/group). Ten rats were designated as the control group fed with a standard diet (Synergetic Bioengineering Co. Ltd., Nanjing, China) throughout the whole experimental period. Fifty rats were fed with an iron-deficient diet (5 mg Fe/kg diet, Synergetic Bioengineering Co. Ltd., Nanjing, China) for 21 days to generate an IDA rat model. After 21 days, the blood of model rats (*n* = 50) was collected by orbital venipuncture into anticoagulant blood vessels for the hematological test, and IDA was defined as hemoglobin (Hb) values below 100 g/L (10). After the IDA rat model was established, the control group was still fed with a standard diet, and 50 IDA rats were randomly subdivided into five groups and still fed with an iron-deficient diet (5 mg Fe/kg/day). Out of five groups, three were administered OP-Fe^2+^ and one FeCl2. The administration was intragastric, once a day for 21 days. The detail dose of each group is shown in Table 2. The ingredients of iron-deficient diet are casein (20%), L-Cystine (3%), corn starch (15%), sucrose (54.999%), ethoxyquin (0.001%), corn oil (5%), mineral mix s18703 (3.5%), choline bitartrate (0.2%), and vitamin mix v1001 (1%).

**Table 2:** Summary of the experiment design and the doses of OP-Fe^2+^ or Fecl_2_ on rats.

**Group**	**Diet**	**Gavage**
Control Group (CG)	Standard diet	Sterile water (5 ml/kg/day)
Model Group (MG)	Iron-deficient diet	Sterile water (5 ml/kg/day)
OP-Fe^2+^ Low Dose Group (OPFL)	Iron-deficient diet	OP-Fe^2+^ (1.0 mg Fe/kg/day)
OP-Fe^2+^ Medium Dose Group (OPFM)	Iron-deficient diet	OP-Fe^2+^ (3.0 mg Fe/kg/day)
OP-Fe^2+^ High Dose Group (OPFH)	Iron-deficient diet	OP-Fe^2+^ (5.0 mg Fe/kg/day)
FeCl_2_ Group (FG)	Iron-deficient diet	FeCl_2_ (3.0 mg Fe/kg/day)

*The above grouping design was carried out after the successful establishment of IDA rat model*.

### Hematological Test

At the end of the experimental period, the rats fasted for 12 h before euthanasia. Blood samples were collected from the abdominal aorta and kept in tubes with an anticoagulant. The serum was separated by centrifugation at 1,850 × g for 15 min and stored at −80°C for further analyses.

The hemoglobin value (HB), number of red blood cells (RBC), mean red cell volume (MCV), and mean red blood cell hemoglobin concentration (MCHC) were measured with an automated blood analyzer (Sysmex F-820, TOA Medical Electronics Co. Ltd., Kobe, Japan). The serum iron (SI) level and total iron and binding capacity (TIBC) were measured using the corresponding assay kits (Nanjing Jiancheng Bioengineering Institute, Nanjing, China). Meanwhile, serum ferritin (SF) was measured by a ferritin assay kit (Nanjing Jiancheng Bioengineering Institute, Nanjing, China). Serum ALT and AST were analyzed by Nanjing Kingmed for Clinical Laboratory using the Beckman AU5800 instrument (Beckman Coulter, Inc., Brea, CA, United States).

### Determination of Antioxidant Activity *in vivo*

The activity of superoxide dismutase (SOD), glutathione (GPX), and malondialdehyde (MDA) levels in liver homogenate were measured with corresponding assay kits (Nanjing Jiancheng Bioengineering Institute, Nanjing, China).

### Organ Coefficient

The spleen and liver were collected and weighed immediately. The liver was rapidly frozen in liquid nitrogen and then stored at −80°C for the next step analysis. The relative weight of each organ was calculated based on the final body weight. The organ coefficient was counted as follows:

$$\begin{array}{l} \text{Organ coefficient }\left(\text{g}/100\text{g}\right)\;=\text{ Organ weight}/\text{rat body weight}\\ \;\times \;100. \end{array}$$

### Statistical Analysis

Data were presented as mean ± SD (*n* = 10). Statistical processing and data analysis were performed using the SPSS software program (SPSS 17.0, SPSS Inc., Chicago, IL, United States) and GraphPad Prism 5.0 (GraphPad Software, San Diego, CA, United States).

One-way analysis of variance (ANOVA) followed by Duncan's new multiple range method was applied to analyze the statistical results. ANOVA data with *p* < 0.05 were considered statistically significant.

## Results

### Results of Single Factor Experiment

**Figure 1:**
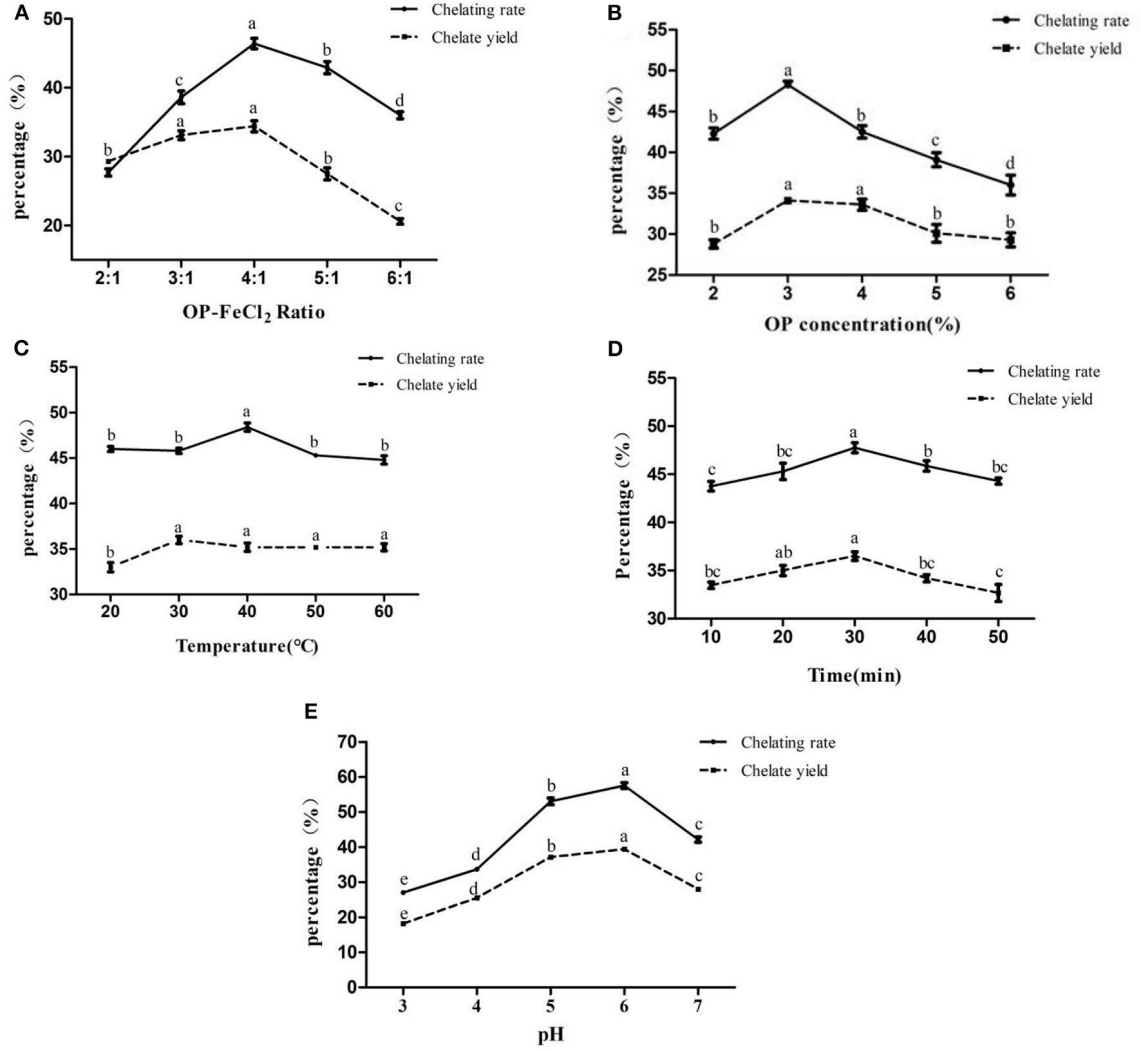
Effects of different binding conditions on chelating rate and chelate yield (**A**:OP-FeCl2 ratio, **B**:OP concentration, **C**: Temperature, **D**: Time, **E**:pH). In the same index, different letters indicate that there are significant differences between different levels (*p* < 0.05), whereas, the same letters mean that there are no statistical differences between different levels (*p* > 0.05).

According to the results of the single factor experiment (Figures 1A–E), the optimal pH, OP-FeCl_2_ ratio, OP concentration, temperature, and time are 6, 4:1, 3%, 40°C, and 30 min, respectively. Three factors (pH, OP concentration, and OP-FeCl_2_ ratio), which have a great influence on the chelating rate, were selected for further response surface optimization experiments.

### Results of Response Surface Methodology Experiment

#### Statistical Analysis and Model Fitting

The chelating rate of OP-FeCl_2_ was optimized by RSM using the Box–Behnken design method. Seventeen combinations were produced by the Box–Behnken design, and each combination was repeated three times, as shown in Table 3. The data of the chelating rate were analyzed by multiple regression to obtain the second-order polynomial equation:

$$\begin{array}{l} \text{R}1=60.68-13.07\text{A}+1.10\text{B}+0.72\text{C}-3.35\text{AB}+8.05\text{AC}\\ \;+4.75\text{BC}-18.72{\text{A}}^{2}-2.52{\text{ B}}^{2}-3.66{\text{C}}^{2} \end{array}$$

where, R1 was chelating rate; A, B, and C represented pH, OP concentration, OP-FeCl_2_ ratio, respectively.

**Table 3:** Central composition test design and results.

**Runs**	**Factors**			**Chelating rate %**
	**A**	**B**	**C**	
1	0	0	0	57.5
2	−1	0	1	46.7
3	1	0	1	36.5
4	−1	−1	0	45.9
5	0	1	−1	50.6
6	−1	1	0	59.0
7	0	0	0	58.5
8	1	1	0	26.3
9	−1	0	−1	56.2
11	0	0	0	62.8
12	1	−1	0	26.6
13	1	0	−1	13.8
14	0	0	0	63.7
15	0	−1	1	48.9
16	0	1	1	56.4
17	0	−1	−1	62.1

The statistical significance of the regression model was tested using F-test and *p*-value. The ANOVA for the response surface quadratic model was summarized in Table 4. The ideal regression equation and the high model significance were confirmed by its low *p*-value (*p* < 0.01) and lack of fit (*p* = 0.1021 > 0.05). The correlation coefficient R2 of the model is 0.9668, indicating that there is a high correlation between the predicted value and the measured value. The results showed that pH value had a significant effect on chelation rate, while the other factors had no significant effect. Among the three factors, the interaction between pH and polypeptide concentration was very significant, while the interaction between the other factors was not significant.

**Table 4:** ANOVA for response surface quadratic model analysis of variance.

**Source**	**Sum of squares**	**df**	**Mean square**	**F value**	***P*-value**
Model	3401.74	9	377.97	22.63	0.0002^**^
A	1367.64	1	1367.64	81.90	<0.0001^**^
B	9.68	1	9.68	0.58	0.4713
C	4.20	1	4.20	0.25	0.6312
AB	44.89	1	44.89	2.69	0.1451
AC	259.21	1	259.21	15.52	0.0056^**^
BC	90.25	1	90.25	5.40	0.0530
A^2^	1474.74	1	1474.74	88.31	<0.0001^**^
B^2^	26.63	1	26.63	1.59	0.2471
C^2^	56.56	1	56.56	3.39	0.1083
Residual	116.90	7	16.70		
Lake of fit	88.37	3	29.46	4.13	0.1021
Pure error	28.53	4	7.13		
Cor total	3518.64	16			

** *extreme significant difference (p < 0.01).*

**Figure 2:**
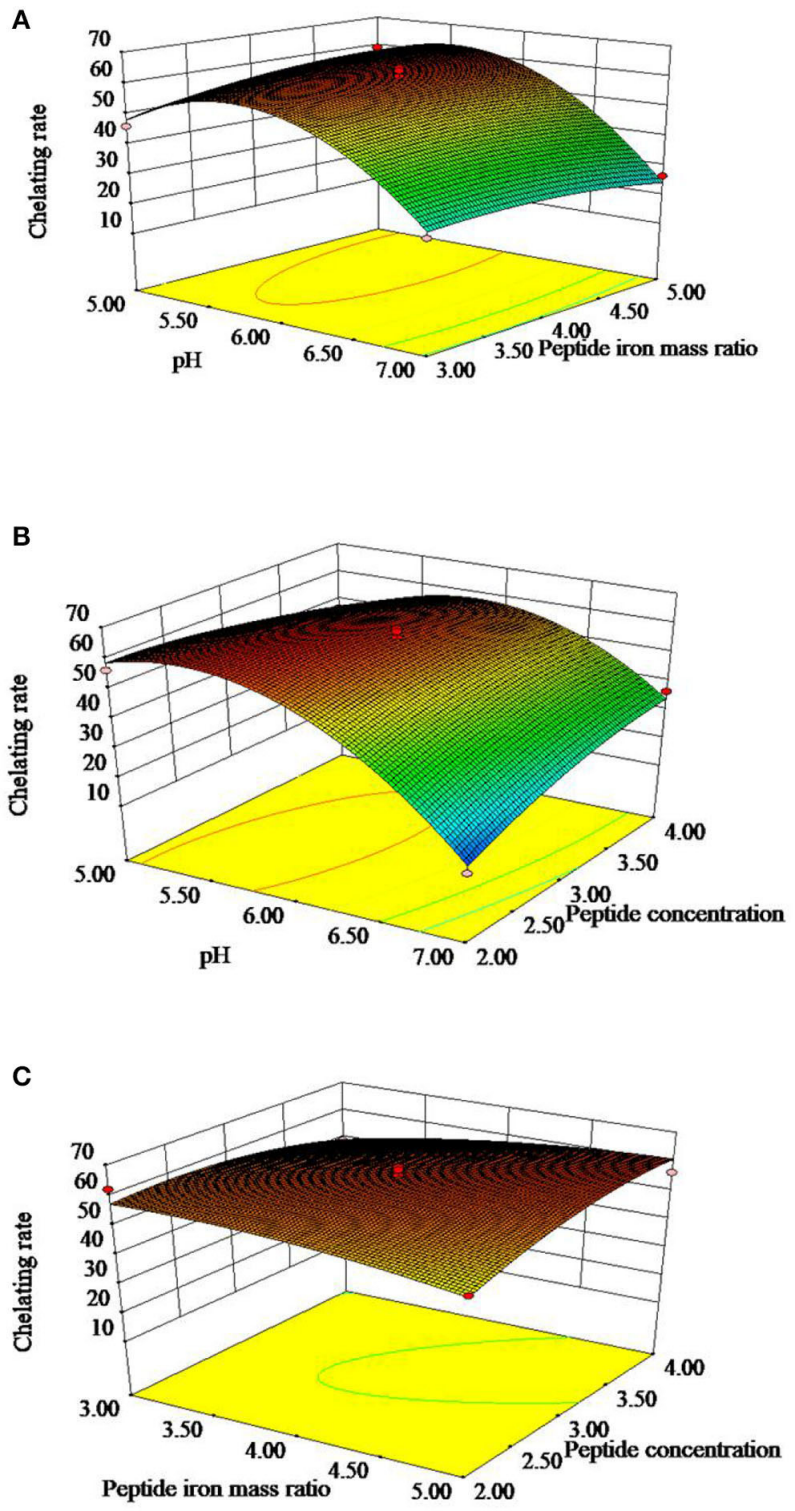
Response surface plots showing effect of peptide concentration, pH, and peptide iron mass ratio on chelating rate of OP-Fe^2+^.

The 3D response surface is shown in Figures 2A–C, which reflected the relationship between two independent variables. Figure 2A represented the effects of OP-FeCl_2_ ratio, pH and their interactions on the chelation rate of OP-Fe^2+^. Figure 2B represented the effects of OP concentration, pH and their interactions on the chelation rate of OP-Fe^2+^. Figure 2C represented the effects of OP concentration, OP-FeCl_2_ ratio and their interactions on the chelation rate of OP-Fe^2+^.

#### Confirmative Tests

The optimum preparation process of OP-Fe^2+^ for maximum chelation rate was obtained as follows: pH = 5.5, peptide:iron = 4.4:1, peptide concentration = 3%, 40°C, 20 min. Under the above conditions, the chelating rate of OP-Fe^2+^ predicted was 63.5%. In order to verify the accuracy of these conditions, the experiment was repeated three times under the above-mentioned conditions, and the actual chelation rate of OP-Fe^2+^ had reached 62.6%. The error different from the theoretical calculation is about 0.9, which indicated that the predicted model could be used for the prediction of the chelating rate of OP-Fe^2+^ totally.

### Determination of Antioxidant Activities

**Figure 3:**
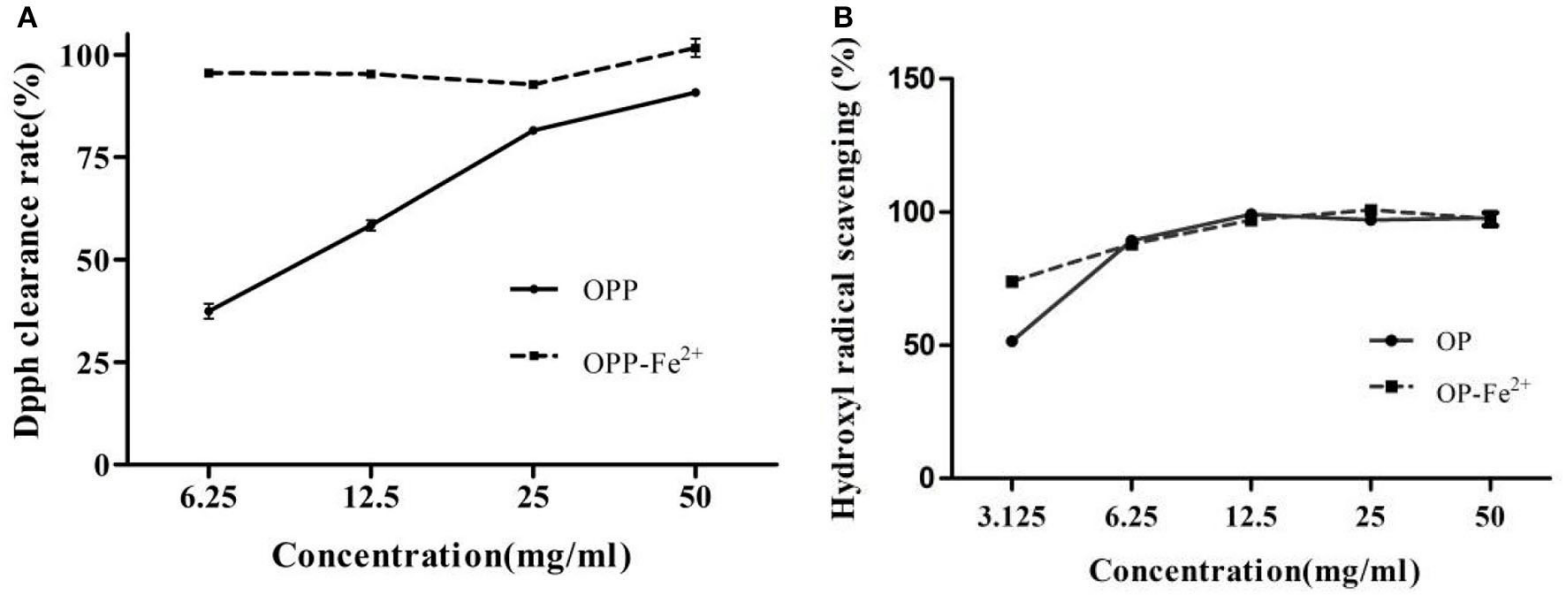
The antioxidant activities of the OP and OP-Fe^2+^ **(A)** DPPH free radical scavenging activity and **(B)** hydroxyl radical scavenging activity.

2,2-diphenyl-1-picrylhydrazyl radical scavenging experiment has extensively been used to evaluate deoxidizing ability (20). As shown in Figure 3A, OP-Fe^2+^ had stronger 2,2-diphenyl-1-picrylhydrazyl (DPPH) radical scavenging activity than OP at the same concentration. The IC_50_ of DPPH radical scavenging rate of OP and OP-Fe^2+^ was 9.11 and 0.02 mg/ml, respectively. It could be discovered from Figure 3B that OP-Fe^2+^ also exhibited stronger activity of scavenging hydroxyl radical than OP at the concentration of 3.125 mg/ml. The IC_50_ of hydroxyl radical scavenging of OP and OP-Fe^2+^ was 3.06 and 1.61 mg/ml, respectively.

### Body Weight Changes in Rats

The changes in the rat body weight on experiment days 0, 28, 35, 42, and 52 are presented in Table 5. When the IDA model was successfully established (28 days), there was a statistical significance (*p* < 0.05) between the CG group and the other groups. After feeding of OP-Fe^2+^ and FeCl_2_ diet for 52 days (administration for 3 weeks and 3 days), no significant differences were observed between the CG group and the other groups except the MG group. These results indicate that OP-Fe^2+^ can supplement the need for iron of the body and reach the normal level.

**Table 5:** The body weight (g) of rats in different groups with time of iron supplementation.

**Group**	**Day of experiment**				
	**0**	**28**	**35**	**42**	**52**
CG	62.62 ± 5.79^a^	291.51 ± 12.24^a^	337.56 ± 18.41^a^	367.65 ± 22.64^a^	375.51 ± 23.78^a^
MG	63.24 ± 4.51^a^	266.29 ± 23.47^b^	289.99 ± 28.45^c^	312.89 ± 31.25^c^	320.43 ± 36.96^b^
OPFL	62.19 ± 4.73^a^	265.31 ± 15.6^b^	302.20 ± 21.95^bc^	336.3 ± 23.86^bc^	359.15 ± 27.97^a^
OPFM	63.39 ± 9.39^a^	254.35 ± 19.28^b^	302.50 ± 22.93^bc^	334.43 ± 23.37^bc^	360.18 ± 25.59^a^
OPFH	60.91 ± 5.17^a^	250.25 ± 14.8^b^	314.88 ± 16.04^ab^	351.01 ± 17.89^ab^	376.99 ± 20.83^a^
FG	65.85 ± 4.73^a^	248.81 ± 19.81^b^	315.50 ± 19.32^ab^	355.46 ± 25.97^ab^	379.71 ± 27.97^a^

*Data are presented as mean ± SD (n = 10). Different letters in the same column indicate a significant difference between different groups (p < 0.05). The same letter in in the same column indicates the absence of a statistical difference between different groups (p > 0.05). 28 d refers to the time when the IDA model was successfully established and has not yet been given treatment; 35 d refers to the second week of treatment with OP-Fe^2+^ and FeCl_2_; 42 d refers to the second week of treatment with OP-Fe^2+^ and FeCl_2_; 52 d refers to the 3 weeks and 3 days of treatment with OP-Fe^2+^ and FeCl_2_*.

### Organ Coefficients of Rats

**Figure 4:**
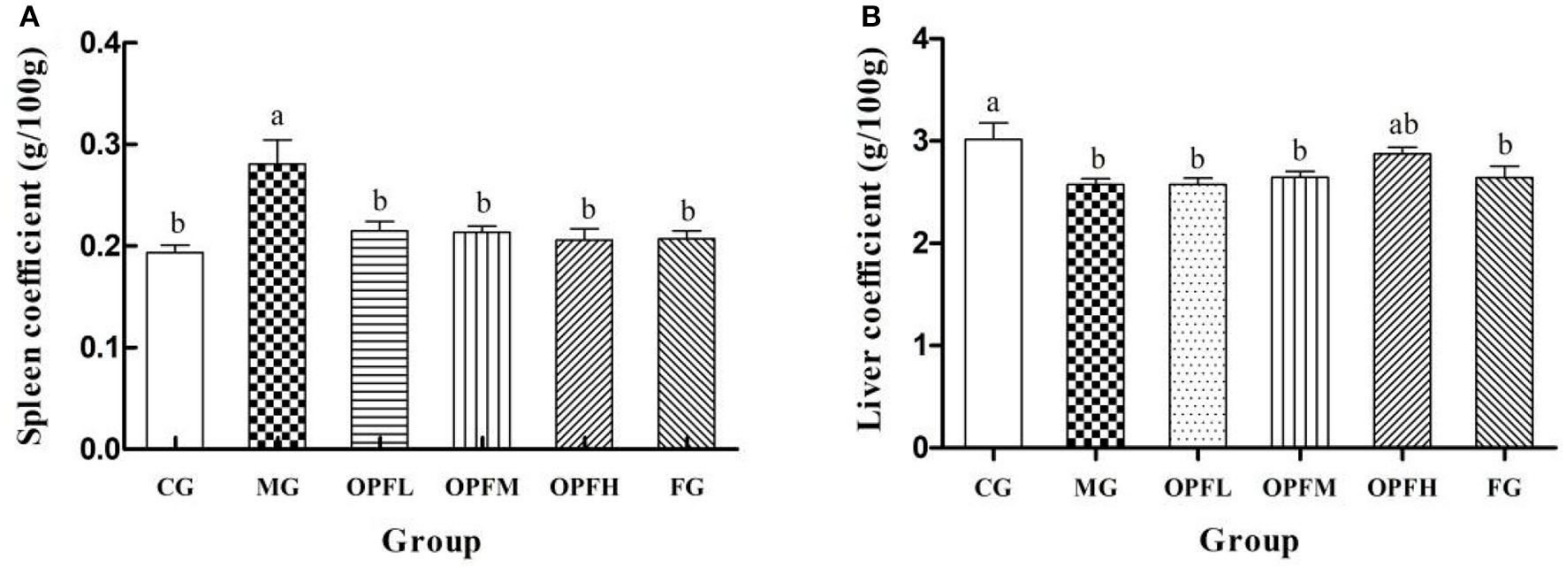
Effects of different doses of OP-Fe^2+^ and FeCl_2_ on **(A)** spleen coefficient and **(B)** liver coefficient in IDA rats. Data are presented as mean ± SD (*n* = 10). The presence of two different letters in two groups indicate a statistical difference (*p* < 0.05) between them; the same letter in two different groups indicates the absence of a statistical difference between these two groups.

The organ coefficients of the rats in each group are shown in Figure 4A. The spleen coefficient levels of the MG group were significantly higher than those of the CG, OPFL, OPFM, OPFH, and FG groups (*p* < 0.05). Meanwhile, there were no significant differences between the CG group and the OPFL, OPFM, OPFH, and FG groups (*p* > 0.05). As shown in Figure 4B, the liver coefficient level of the MG group was significantly lower than that of the CG and OPFH groups (*p* < 0.05). No significant differences were found between the CG and OPFH groups (*p* > 0.05). These results indicated that OP-Fe^2+^ could relieve hypertrophy of the spleen that was caused by anemia.

### Hematological Analysis

**Figure 5:**
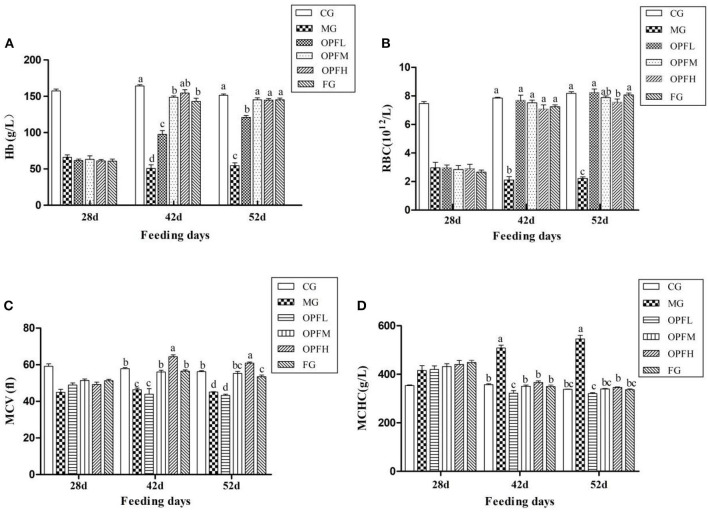
Effect of different doses of OP-Fe^2+^ and FeCl_2_ on **(A)** Hb, **(B)** RBC, **(C)** MCV, and **(D)** MCHC in IDA rats. Different letters indicate a significant difference between different groups in the same period (*p* < 0.05). The same letter in different groups indicates the absence of a statistical difference between these groups in the same period (*p* > 0.05). 28 d refers to the time when the IDA model was successfully established and has not yet been given treatment; 42 d refers to the second week of treatment with OP-Fe^2+^ and FeCl_2_; 52 d refers to the 3 weeks of treatment with OP-Fe^2+^ and FeCl_2_.

Hematological indices, such as Hb, RBC, MCV, and MCHC were measured, which could reflect the situations of iron deficiency. In the MG group, the level of Hb, RBC, and MCV were lower than that in the CG group (Figures 5A–C), *p* < 0.01), suggesting that the model was built as expected successfully. OP-Fe^2+^treatment can significantly improve this change. After treatment with OP-Fe^2+^ for 2 weeks, the Hb levels in the OPFM and OPFH groups were significantly increased by 65.9 and 67.1%, respectively, when compared with the MG group (Figure 5A, *p* < 0.01). As shown in Figure 5B, the RBC level of each treatment group reached the normal level (*p* > 0.05). There were no significant differences in MCV levels between the CG and OPFM groups and the FG group (Figure 5C, *p* > 0.05) after OP-Fe^2+^ treatment for 2 weeks. As shown in Figure 5D, after 3 weeks, each treated group reached the normal MCHC level. The results of hematological indices indicated that OP-Fe^2+^ could improve the hematological indices of rats caused by anemia.

### Serum Iron, Total Iron Binding Capacity, and Serum Ferritin Level of Rats

**Figure 6:**
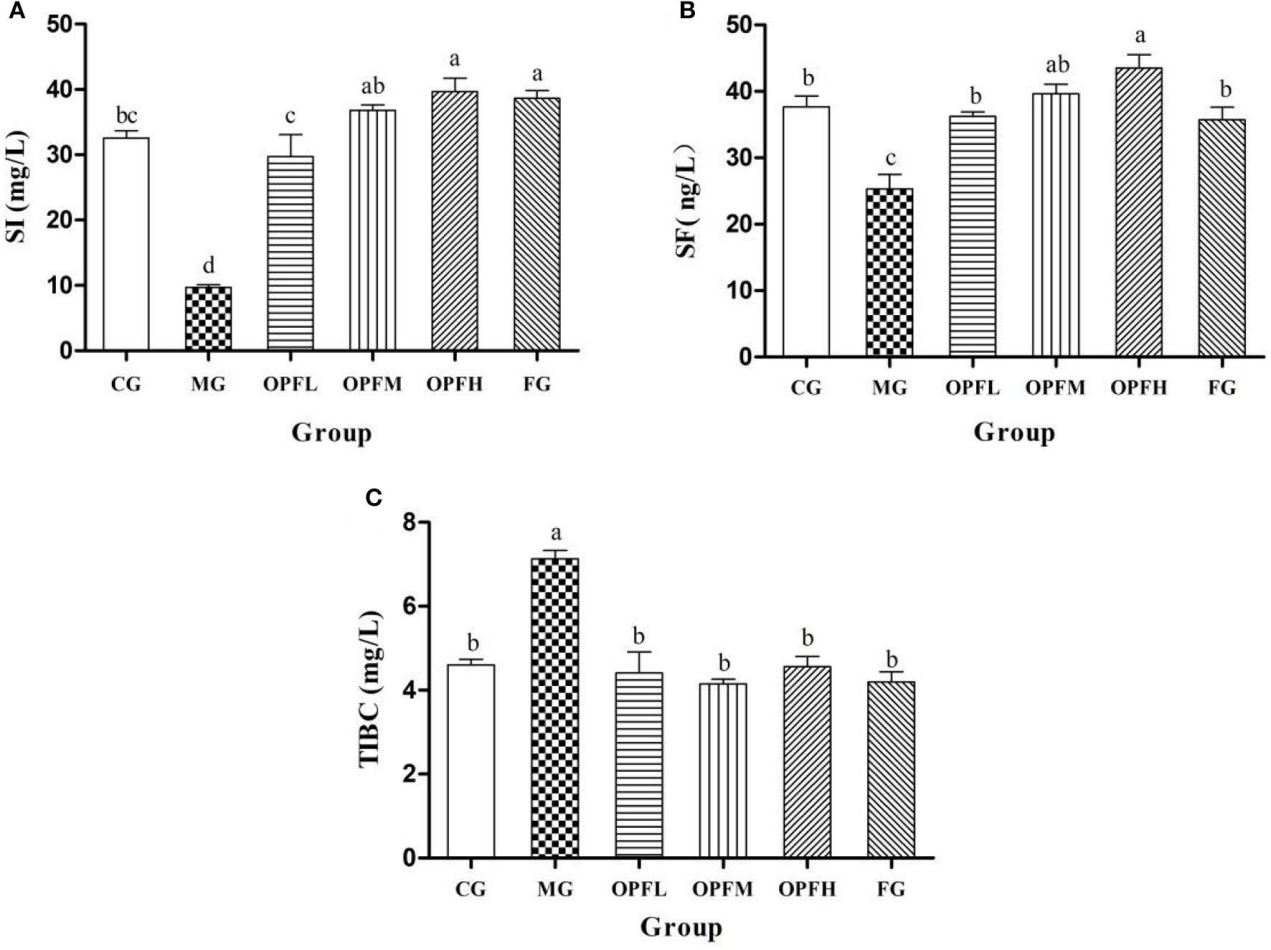
Effect of different doses of OP-Fe^2+^ and FeCl_2_ on **(A)** SI, **(B)** SF, and **(C)** TIBC in IDA rats. Data are presented as mean ± SD (*n* = 10). Different letters show statistically significant differences (*p* < 0.05). The same letter in different groups indicates the absence of a statistical difference between these groups in the same period (*p* > 0.05).

Serum iron, iron binding capacity, and serum ferritin levels are useful parameters for the clinical diagnosis of iron deficiency anemia. In the MG group, the content of serum iron (SI) and serum ferritin (SF) was significantly lower than that in the CG and FG groups [(Figures 6A, 5B), *p* < 0.01], Then, the SI level increased after OP-Fe^2+^ treatment, and there is no significant difference between the OPFL, OPFM, and CG groups (*p* > 0.05). As shown in Figure 6B, no significant differences in SF results were found between the CG group and the OPFL, OPFM, and FG groups (*p* > 0.05). The data of TIBC showed that the MG increased by 54.3% when compared with the CG group (Figure 6C, *p* < 0.01). However, OP-Fe^2+^ treatment obviously inhibited the change, and each treatment group reached the normal TIBC content. These results showed that OP-Fe^2+^ could effectively recover SI, SF, and TIBC levels in rats.

### Analysis of Antioxidant Activity *in vivo*

**Figure 7:**
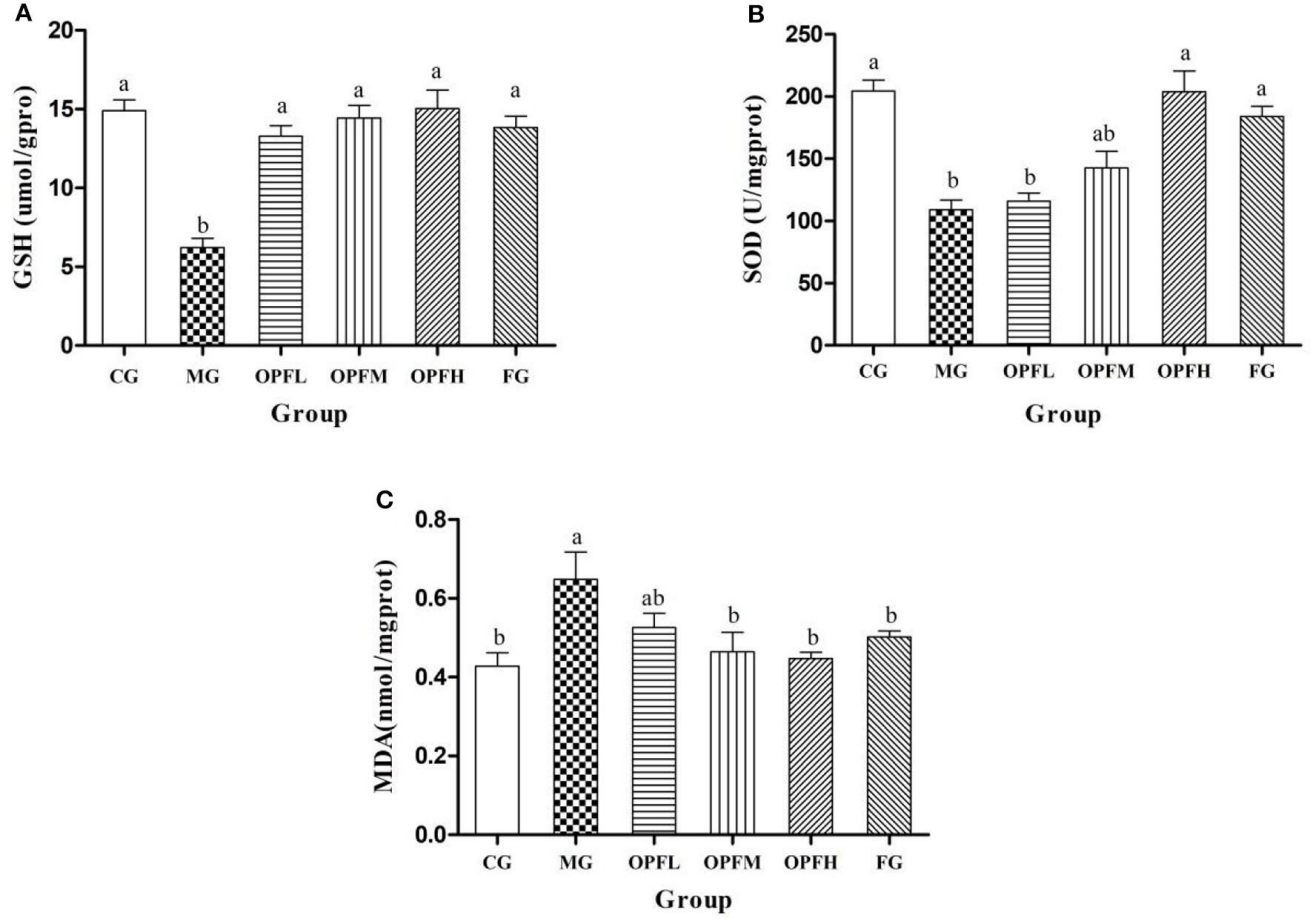
Effects of different doses of OP-Fe^2+^ and FeCl_2_ on antioxidant activity in liver. **(A)** GSH content, **(B)** SOD activity, and **(C)** MDA content. Dates are presented as mean ± SD (*n* = 10). The presence of different letters in different groups indicate a statistical difference (*p* < 0.05) between them; the same letter in different groups indicates the absence of a statistical difference between these groups (*p* > 0.05).

GSH is a type of antioxidant peptide in organisms, which can reflect the antioxidant capacity of the body. As shown in Figure 7A, the GSH activity of the OPFL, OPFM, OPFH, and FG groups was significantly higher than that of the MG group (*p* < 0.05), and there was no significant difference when compared with the CG group (*p* > 0.05). Furthermore, no significant difference was observed between the OPFL and FG groups (*p* > 0.05). SOD is an important antioxidant enzyme, and it could prevent damage produced by free radicals (21). As shown in Figure 7B, the SOD activity of rats in the MG group was significantly lower (*p* < 0.05) than that of the CG group. Meanwhile, the SOD activities of the OPFH and FG groups had no significant differences when compared with the CG group (*p* > 0.05).

Malondialdehyde is a lipid peroxidation metabolite. It can generally serve as an indicator of a generation of free radicals and of damage to the membrane lipid bilayer (22). As shown in Figure 7C, compared with the CG group, iron deficiency markedly increased MDA content in liver homogenate (*p* < 0.05). After OP-Fe^2+^ supplementation, the MDA content of the OPFM and OPFH groups was significantly lower than that of the MG group (*p* < 0.05), and it returned to the normal level.

### Liver Function Test

**Figure 8:**
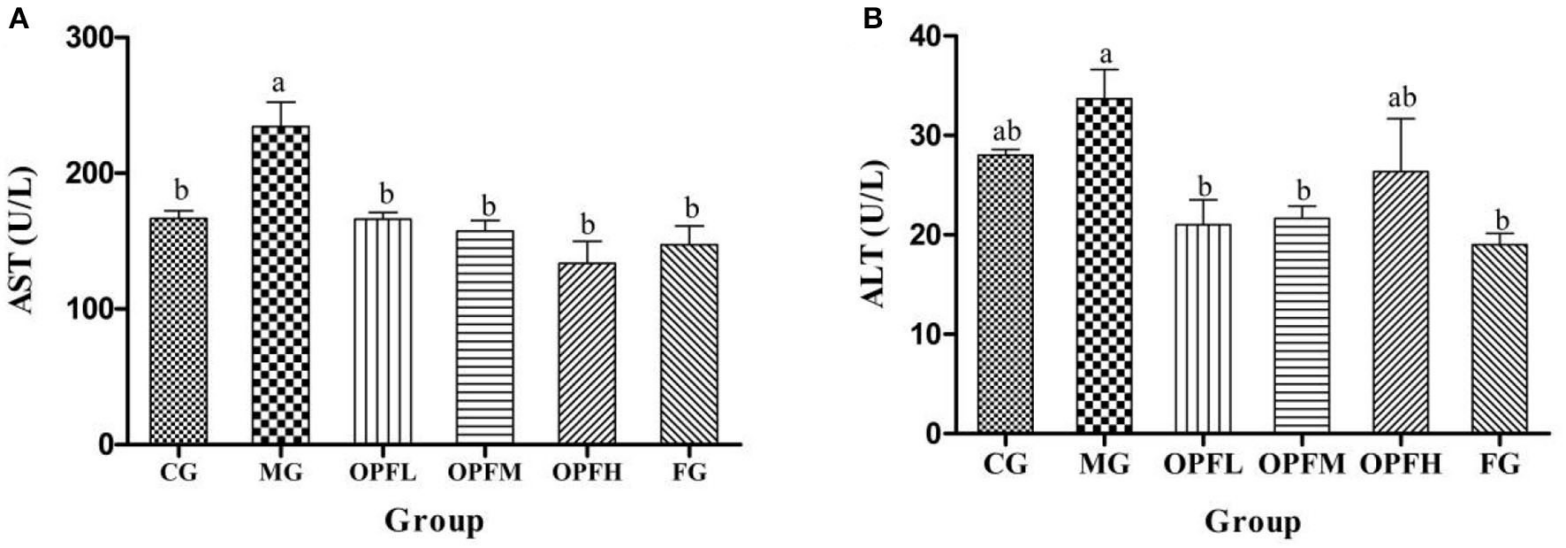
Effects of different doses of OP-Fe^2+^ and FeCl_2_ on **(A)** AST and **(B)** ALT levels in serum. Different letters show statistically significant differences (*p* < 0.05). The same letter in different groups indicates the absence of a statistical difference between these groups (*p* > 0.05). The same is true in ALT.

The evaluation of ALT and AST is utilized as a significant diagnostic marker to indicate liver injury. As is shown in Figure 8, the content of AST in the MG group was higher than that in the CG and FG groups (*p* < 0.01). After OP-Fe^2+^ treatment, the ALT level was decreased, and the ALT level in the OP-Fe^2+^and FG groups did not significantly differ from that in the CG group (*p* > 0.05).

## Discussion

According to the Global Burden of Disease Study 2016, iron deficiency anemia is one of the five leading causes of years lived with disability burden. A worldwide survey showed that anemia has affected one-third of the population, and in 2010, approximately half of the cases resulted from iron deficiency (4). Iron deficiency anemia rarely causes death, but it is harmful to human health. Although, several iron supplements have been in practice for the prevention of anemia since the past decades, there are still some problems, such as low bioavailability and side effects, which limit their clinical application. There is a need for an effective strategical approach that runs for a long time. Iron fortification in foods is a promising and cost-effective approach in targeting a particular group of people for the long run (23).

Food-derived bioactive peptides could be released from their native protein *via* enzymatic processing, which has the advantages of easy absorption and good biological activity. Meanwhile, some bioactive peptides prepared by the enzymatic method soften have a strong ability to chelate metals, and they can effectively deliver metals across the epithelial wall (24). Therefore, this study aimed to synthesize a new type of OP-Fe^2+^ using OP as the carrier, and then the animal experiment was designed to evaluate the iron supplement effect of OP-Fe^2+^ on iron deficiency anemia (IDA) in rats. It has been reported that rats with IDA had low hemoglobin, low serum ferritin, and high TIBC level (25). In this study, we gained the same characters on the IDA model group. Then, after the application of OP-Fe^2+^ diet, no significant differences in these parameters were observed between the normal group and the treated groups, which means that the OP-Fe^2+^ chelate had good iron supplement effects on the rats with IDA. Furthermore, the body weight changes and the organ coefficients results also indicated that the supply of OP-Fe^2+^ chelate could improve the body damage caused by IDA.

Iron is helpful to maintain homeostasis and regulate a wide variety of physiologic and metabolic pathways, such as oxygen transport, oxidative phosphorylation, and many other enzymatic pathways (26, 27). Besides, the dysregulation of iron pathways could lead to cellular oxidative stress (28). Oxidative stress is mainly attributed to the generation of hydroxyl radicals and the initiation of lipid peroxidation. Lipid peroxidation creates mutagenic reactants, such as aldehyde malondialdehyde (MDA) (27). Hydroxyl and superoxide anion radicals can easily react with carbohydrates, proteins, and DNA, leading to oxidative stress, cell injury, and physiological disorders, all damages of which are harmful to the human body (12). Antioxidant molecules have been considered as a strategy to prevent or reduce the incidence of many health-related conditions (29). It was reported that the antioxidant activity of chelates is higher than that of peptides. Lin et al. (30) pointed out that the free radical scavenging activity of four low-value fish hydrolyzed peptides was weak, but 5 mg/ml hydrolyzed protein ferrous chelating peptides showed strong activity of scavenging DPPH radicals. In this study, for the *in vitro* antioxidant activity assays, OP-Fe^2+^ showed stronger scavenging effects on hydroxyl radicals and DPPH than oat peptides, and the results showed that OP-Fe^2+^ enhanced the antioxidant activity of OP. In addition, SOD and glutathione (GSH) are important factors in the prevention of oxidative stress through their action on reactive oxygen species (ROS) (21). In animal experiments, compared with those of anemic rats without OP-Fe^2+^ supplementation, the activities of SOD and GSH in rats of OP-Fe^2+^ treated groups significantly increased. Moreover, OP-Fe^2+^ significantly reduced the MDA content in the liver of rats. Besides, AST results showed that OP-Fe^2+^ could alleviate the hepatic injury caused by iron deficiency. These results suggested that OP-Fe^2+^ was helpful in restoring the antioxidant capacity of anemic rats.

Though iron is biologically essential, its overload has potential toxicity including oxidative damage (31). In this study, we used the OPFL, OPFM, and OPFH groups treated with OP-Fe^2+^ to investigate the supplementary effect on iron deficiency in the IDA model. The results indicated that the OP-Fe^2+^ of three different doses had good functions of iron supply, and no overload effect was detected. There are no significant side-effects on the OP-Fe^2+^-treated group. We speculated that there are two probable reasons. The first one is the structure of OP-Fe^2+^, a special combination of the oat peptides and iron, which regulates OP-Fe^2+^ transportation and keeps iron absorption to meet the requirement of the body. The second reason may exist in the self-regulation of iron absorption, storage, and metabolism. The detailed regulatory process needs to be studied in the future. In addition, the molecular mechanism of the active application of OP-Fe^2+^ chelate will be an interesting research direction, including the association between the OP-Fe^2+^ and ROS and immune (32–34).

## Conclusion

In this study, the preparation technology of OP-Fe^2+^ was optimized. After chelating with iron, the antioxidant capacity of oat peptides was significantly enhanced. The animal experiment indicated that OP-Fe^2+^ can be used as an effective iron supplement and antioxidant. The regulation mechanism of OP-Fe^2+^ in the restoration of the antioxidant capacity in rats is still unclear. Further studies are necessary to be conducted.

## Data Availability Statement

The original contributions presented in the study are included in the article/supplementary material, further inquiries can be directed to the corresponding author/s.

## Ethics Statement

The animal study was reviewed and approved by Institutional Animal Care and Use Committees of Jiangsu University (UJS-IACUC-2020072201).

## Author Contributions

HY and YP contributed to conception and design of the study and wrote the first draft of the manuscript. HY organized the database. HY and DYa embellished the article. YP, CM, DYu, and GR performed the statistical analysis. YP and CM carried out specific experiments. HY, DYa, ZH, MH, and DYu wrote sections of the manuscript. All authors contributed to manuscript revision, read, and approved the submitted version.

## Conflict of Interest

The authors declare that the research was conducted in the absence of any commercial or financial relationships that could be construed as a potential conflict of interest.
